# Bacillary hemoglobinuria in a dairy cow in Goiás State, Central-West Brazil, without demonstrable evidence of active fascioliasis

**DOI:** 10.1007/s11259-026-11537-1

**Published:** 2026-09-26

**Authors:** Filipe Augusto Vieira dos Santos, Isadora David Tavares de Moraes, Tamires Ataides Silva, Warley Vieira de Freitas Paula, Carmen Elena Barragan-Ruiz, Noenia Sacramento Rocha, Felipe da Silva Krawczak, Mariana Pires de Campos Telles, Ana Carolina Borsanelli, Fabiana Marques Boabaid, Luiz Gustavo Schneider de Oliveira

**Affiliations:** 1 Department of Veterinary Medicine, Escola de Veterinária e Zootecnia, Universidade Federal de Goiás, Campus Samambaia, Rodovia Goiânia-Nova Veneza, km 8, Goiânia, 74690-900 Goiás Brazil; 2 Universidade Estadual de Goiás, Campus Oeste, São Luís dos Montes Belos, Goiás, Brasil; 3 Institute of Biological Sciences, Universidade Federal de Goiás, Goiânia, Goiás Brazil

**Keywords:** *Clostridium haemolyticum*, *Clostridium novyi* type D, Cattle diseases

## Abstract

In a dairy herd in the state of Goiás, Central-West Brazil, a cow died following an acute illness characterized by jaundice and hemoglobinuria. Necropsy revealed jaundice, dark-red urine and dark-red discoloration of the kidneys, and a diffusely copper-colored liver with two extensive, firm lesions surrounded by hyperemic halos in the right and left lobes. Histopathological analysis showed multifocal coagulative necrosis in the liver, accompanied by abundant intralesional bacillary structures and multifocal thrombosis. Renal lesions included tubular cell degeneration and necrosis, intracellular hyaline droplet accumulation, and occasional proteinaceous casts within the tubular lumen. PCR and sequencing confirmed the presence of *Clostridium haemolyticum* DNA in the liver lesions. Based on the characteristic clinical presentation and pathological findings, the diagnosis of bacillary hemoglobinuria was established, with further molecular evidence of the clostridial agent. To our knowledge, this is the first reported case of bacillary hemoglobinuria in the Central-West region of Brazil, and an unusual report in the Tropical Zone in the country, especially in dairy cattle.

## Background

Bacillary hemoglobinuria is an acute and highly lethal infectious disease of cattle, which occasionally affects sheep, pigs, horses, and elk (Navarro et al. 2016). Bacillary hemoglobinuria is caused by *Clostridium haemolyticum*, also known as *Clostridium novyi* type D, a spore-forming, motile, gram-positive and anaerobic bacillary bacterium. The main virulence factor of this pathogen is β-toxin, a necrotizing and highly hemolytic phospholipase C, which causes endothelial rupture and hepatocyte necrosis (Navarro et al. 2016, 2017).

In temperate and subtropical environments, the disease is reported mainly in summer and fall, in regions where the parasitism by liver flukes (*Fasciola hepatica*) is endemic in cattle, corresponding to pastures localized in floodplains with poor drainage and alkaline soils (Radostits et al. 2002; Barros 2016). Pasture contamination by *C. haemolyticum* spores can result from fecal or urinary elimination by healthy cattle reservoirs or from decomposing carcasses (Barros 2016; Navarro et al. 2016). The spores are highly resistant to environmental conditions and can be dispersed to different areas via flooding, natural drainage and vectors (Barros 2016; Dutra et al. 2022).

Spores may be ingested with feed, being absorbed through the intestine, and reach the liver via portal circulation, where they remain latent in Kupffer cells, until conditions become favorable for germination (Barros 2016; Navarro et al. 2016). Larval migration of *F. hepatica* or other trematodes, such as *Fascioloides magna*, through the liver parenchyma can induce lesions that lead to anaerobic conditions, permitting the spores to germinate and produce β-toxin. It is believed that combined effects of β-toxin activity and thrombosis-induced hypoxia lead to extensive foci of hepatic coagulative necrosis. After reaching the systemic circulation, the toxin causes hemolysis, resulting in severe anemia, hemoglobinuria, and jaundice, usually culminating in death (Navarro et al. 2016; Ierardi et al. 2025).

Although the disease is considered endemic in certain geographical locations, its occurrence is widely distributed in many countries of South and North America, Asia, Europe and Oceania (Radostits et al. 2002; Navarro et al. 2016; Dutra et al. 2022). In Brazil, bacillary hemoglobinuria is considered endemic in the Southern states, especially on poorly drained landscapes, which support the existence of *Pseudosuccinea* (*Lymnaea*) spp. snails, the first intermediate host of *F. hepatica* (Schild 2007; Raymundo et al. 2014).

Nevertheless, bacillary hemoglobinuria can occasionally be seen in regions where *F. hepatica* does not occur. In such cases the initial hepatic lesion can be associated with a variety of other agents (Janzen et al. 1981; Radostits et al. 2002; Barros 2016; Dutra et al. 2022). In our study, we report the occurrence of bacillary hemoglobinuria in the state of Goiás, in Central-West Brazil. It also represents one of the few reports of bacillary hemoglobinuria affecting a dairy cow, which had no gross and microscopic findings compatible with *F. hepatica* parasitism in the liver.

## Case presentation

In January 24, 2024, the Animal Pathology Department of the Federal University of Goiás (UFG) conducted a visit to a dairy farm located in the Central-West of Brazil, in the municipality of Bela Vista de Goiás, in Goiás state, to perform a necropsy in a cow from a herd of 59 cattle, including Gir, Holstein and Girolando breeds. According to the owner, the herd was composed of 34 cows, which were housed outdoors part-time and fed grass, corn silage, and a mixed ration. The owner stated that at the beginning of December of 2023, the herd had been joined by 25 new individuals from different sources. No quarantine period was established, and the newly introduced animals were immediately integrated into the same management practices as the rest of the herd. This included the administration of injectable drugs and vaccines using the same needle across multiple animals. Following the introduction of the new cows, at the end of December of 2023, the farmer noted the onset of several health issues and the mortality of 17 cows within a short period, including cows born at the farm and recently introduced ones. The initial clinical signs included cases of mastitis and keratoconjunctivitis in different cows, which were treated by a veterinarian. Despite medical assistance, many cows developed severe apathy, loss of appetite, as well as abortions and diarrhea. Additionally, the attending veterinarian reported that ten of the affected cows tested positive for circulating antibodies against *Trypanosoma vivax* through chromatographic immunoassay. According to the farmer, after January 24, 2024, six additional cows died until the end of February, when the outbreak finally stopped. The epidemiological data are part of the sanitary register of the farm presented by the owner, based on the previous referring veterinary assistance. In Table 1a summary of the epidemiological events are presented in a chronological manner.

**Fig. 1:**
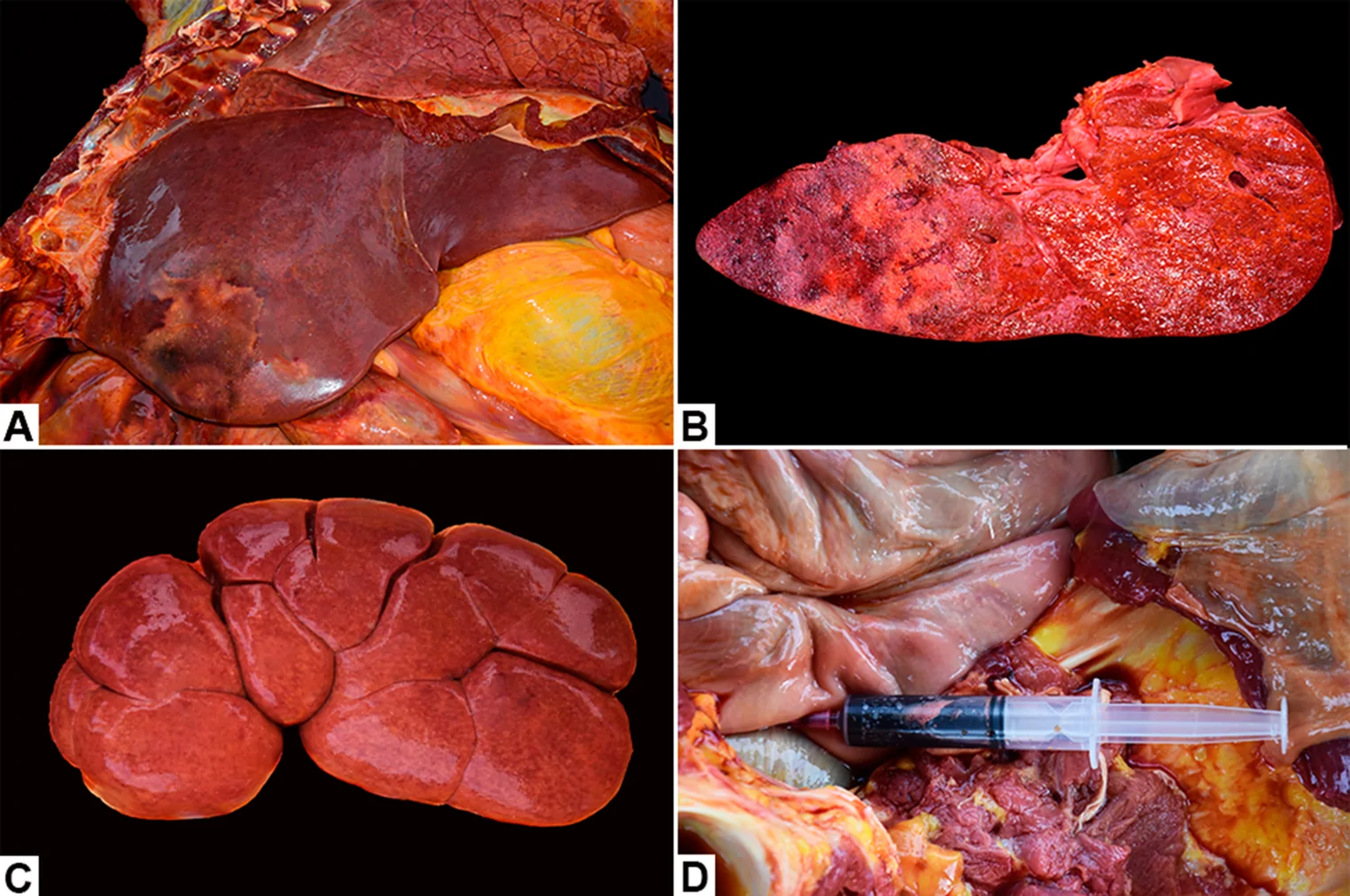
Bacillary hemoglobinuria in a dairy cow. (**A**) Note the diaphragmatic aspect of right liver lobe with a large pale tan area delimited by a hyperemic halo and covered with a thin layer of fibrin. (**B**) The cut surface of the right lobe of the liver reveals the pale tan foci extending into the parenchyma with a marbling appearance; the remaining liver has an orange-brown color. (**C**) The kidney exhibits multifocal disseminated 1–2 mm dark-red dots on the cortical surface. (**D**) A syringe with a needle was introduced into the urinary bladder to demonstrate the dark-red content

A necropsy was performed on an adult Girolando cow in fair body condition, in the second trimester of gestation. On external examination the ocular conjunctivae, as well as the oral and vulvar mucosae, were markedly icteric. The subcutaneous tissue was similarly icteric and exhibited multifocal extensive hemorrhage and edema. Also, the omentum, mesentery, and serosae of the abdominal organs were moderately icteric. On the diaphragmatic surface of both the right and left liver lobes, there were focal extensive, well-demarcated, pale tan areas, delimited by a hyperemic halo (Fig. 1A). These necrotic foci extended into the hepatic parenchyma (Fig. 1B), were firm and raised, and the capsular surface was covered with a thin fibrin mesh. The right lobe lesion dimensions were of ca. 35 cm x 30 cm x 25 cm, and was located next to the right triangular ligament, visible on both diaphragmatic and visceral surfaces, while the left lobe lesion measured ca. 25 cm x 20 cm x 20 cm and was located at the border, visible on both diaphragmatic and visceral surfaces. The remaining liver parenchyma was diffusely orange-brown. The kidneys exhibited multifocal disseminated 1–2 mm dark-red foci on the cortical surface (Fig. 1C). The urinary bladder contained dark-red urine (Fig. 1D). The spleen was enlarged, with rounded borders and protruding the red pulp at cut surface. The thoracic cavity contained a moderate amount of serous yellowish fluid. The lungs exhibited expanded interlobular septa with a gelatinous aspect, consistent with interlobular edema. The remaining organs had no significant gross findings. Tissue sections were fixed in 10% buffered formalin and processed routinely for histologic evaluation.

**Fig. 2:**
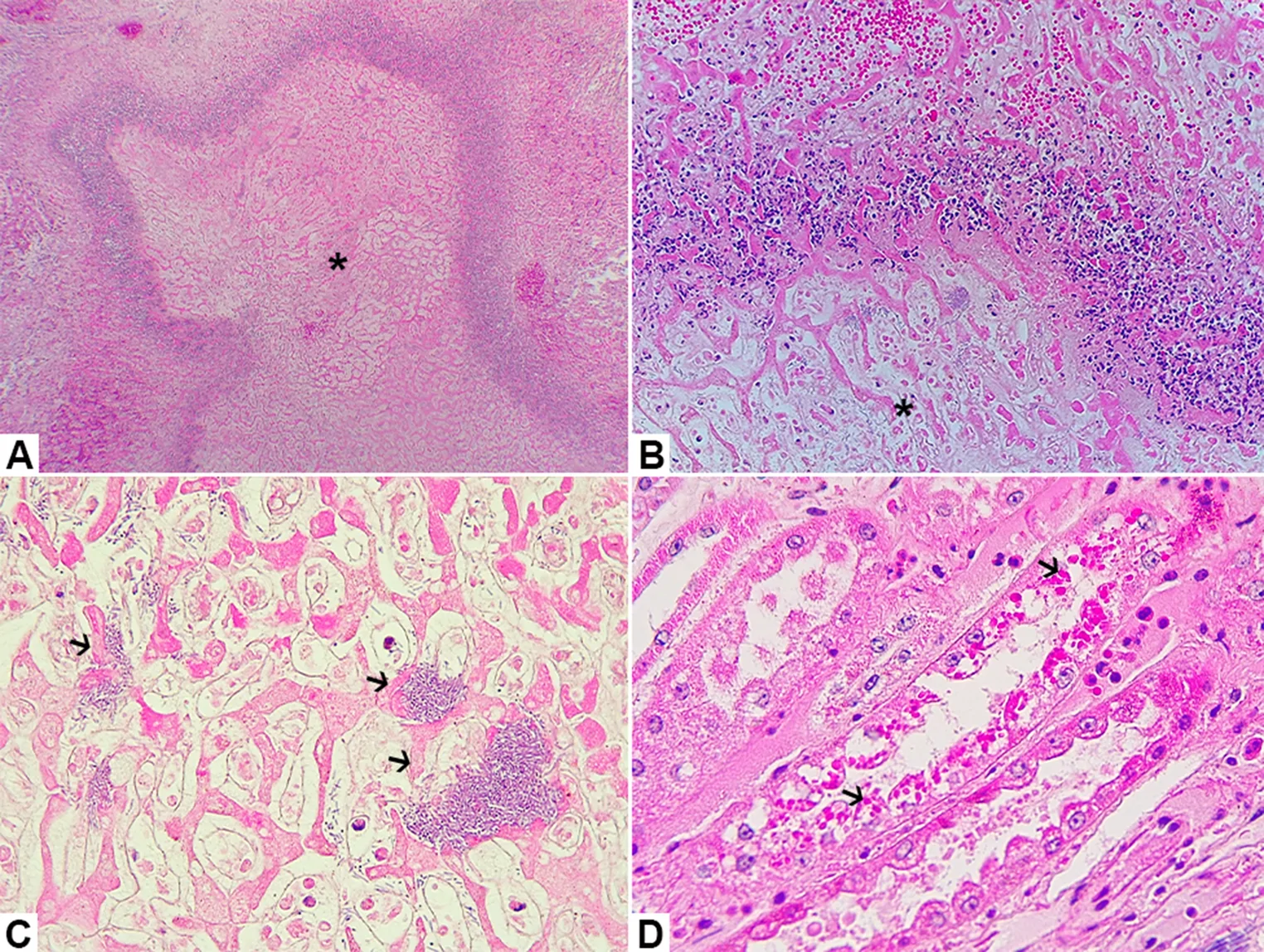
Bacillary hemoglobinuria in a dairy cow. (**A**) Liver, a focally extensive area of coagulative necrosis (*) is sharply delimited from the rest of the liver (HE, x4). (**B**) Closer view of the hepatic necrotic focus (*), exhibiting a rim of inflammatory cells, mainly composed by neutrophils. At the inner margin of the inflammatory rim are multifocal clusters of bacterial structures (HE, x20). (**C**) At the coagulative necrotic foci are multifocal clusters of bacillary bacteria between the hepatic cords (arrows) (HE, x40). (**D**) Kidney, cortex, the tubular epithelial cells contain abundant intracytoplasmic hyaline droplets (arrows) (HE, x40)

Upon histological examination, the liver had a focal extensive, well-demarcated area of coagulative necrosis, surrounded by a multifocal severe infiltrate of degenerate neutrophils and fewer macrophages, lymphocytes and plasma cells (Fig. 2A and B). Inside the sinusoids of the necrotic foci were numerous basophilic bacillary structures, forming massive aggregates, especially along the margin of the inflammatory halo (Fig. 2C). Large blood vessels multifocally contained massive thrombi. The remaining liver had multifocal moderate hepatocellular degeneration, characterized either by feathery cytoplasmic vacuolization or large vacuoles pushing the nucleus to the periphery. Furthermore, there was a multifocal severe periportal infiltrate of lymphocytes and plasma cells. In the kidney, there was multifocal mild tubular degeneration and necrosis, as well as multifocal intracellular variably sized intracytoplasmic hyaline droplets (Fig. 2D) and occasional proteinaceous casts filling the tubular lumen. Additionally, multifocal mild inflammatory infiltrate of lymphocytes and plasma cells was noted at the interstitial space.

A fresh sample of the liver lesion was submitted for molecular detection of *Clostridium* species at the Laboratory of Infectious Diseases and Bacteriology. Genomic DNA was extracted from the liver sample using the DNeasy^®^ Blood and Tissue kit (Qiagen, Valencia, CA, USA) following the manufacturer’s instructions. The presence of *Clostridium haemolyticum*,* C. septicum* and *C. chauvoei* in the liver sample was assessed by conventional polymerase chain reaction (cPCR) using specific primers (Sasaki et al. 2002).

Concomitantly, fresh spleen samples were submitted for molecular detection, and DNA was extracted using the same kit previously described. Subsequently, cPCR assays were performed for the detection of *Anaplasma marginale*, *Babesia bovis*, *Babesia bigemina*, and *T. vivax*. DNA samples were subjected to PCR protocols targeting a 177 bp fragment corresponding to the catalytic domain of CatL-like (*cdCatL-like*) sequences of *T. vivax* (Cortez et al. 2009). Additional assays targeted a 458 bp fragment of the major surface protein 5 (*msp5*) gene of *A. marginale* (De Echaide et al. 1998), a 356 bp fragment of the rhoptry-associated protein 1a (*Rap-1a*) gene of *B. bovis* (Parodi et al. 2021), and an approximately 440 bp fragment of the variant erythrocyte surface antigen (*ves-1α*) gene of *B. bigemina* (De La Fournière et al. 2021). Negative (PCR-grade water, Sigma-Aldrich, St. Louis, MO, USA) and appropriate positive controls (DNA from *B. bovis*, *B. bigemina*, *A. marginale*, and *T. vivax*) were included in all assays.

The fragment of the liver lesion tested positive for *C. haemolyticum* by PCR. Amplification for sequencing purposes was performed using the primer pair 27 F (AGAGTTTGATCCTGGCTCAG) and 534R (ATTACCGCGGCTGCTGG), which targets the V1–V3 regions of the bacterial 16 S rDNA, generating an amplicon of approximately 500 bp. Each PCR reaction was prepared in a final volume of 10 µL, containing 1.7 µL of molecular-grade water, 1.3 µL of BSA (25 mg/mL), 0.9 µL of dNTPs (2.5 µM), 1.0 µL of 10× PCR buffer containing MgCl_2_, 1.3 µL of forward primer (1 µM), 1.3 µL of reverse primer (1 µM), 0.5 µL of Taq DNA polymerase (5 U), and 2.0 µL of template DNA (2,5ng/µL). The PCR thermal profile consisted of an initial denaturation at 94 °C for 2 min, followed by 40 cycles of denaturation at 94 °C for 1 min, annealing at 55 °C for 30 s, and extension at 72 °C for 1 min. A final extension step was performed at 72 °C for 10 min, after which the samples were maintained at 4 °C. The PCR products were visualized by horizontal gel electrophoresis. Sequencing was performed in both directions, forward and reverse, by capillary electrophoresis using an Applied Biosystems 3500 Series Genetic Analyzer. The sequences obtained were analyzed and assembled using Geneious R6.0 software. Bacterial species identification was conducted by comparison with reference sequences available in the National Center for Biotechnology Information (NCBI) database (Sayers et al. 2022). The resulting sequences showed high similarity with *C. haemolyticum.* In conjunction with the species-specific PCR results and the pathological findings, these findings were considered consistent with the identification of *C. haemolyticum*. Spleen samples tested positive for *A. marginale*, and negative for *B. bovis*, *B. bigemina* and *T. vivax* by cPCR.

Additionally, fecal samples from eight affected cows were analysed using the sedimentation test for the detection of *Fasciola hepatica* eggs (Monteiro 2017), performed at the Laboratory of Parasitic Diseases (LADOPAR). All samples collected from these cows tested negative for *F. hepatica* eggs.

**Table 1:** Chronological summary of the main sanitary events reported on the farm

Date/Period	Event
Early December 2023	A herd of 34 cows (Gir, Holstein, Girolando) received 25 newly introduced cows from different sources; no quarantine period was applied
Late December 2023	Onset of health problems in the herd, including mastitis, keratoconjunctivitis abortions, diarrhea, apathy, anorexia, and the first deaths reported
Early January 2024	Ten affected cows tested positive for anti-*Trypanosoma vivax* antibodies by chromatographic immunoassay
January 24, 2024	A total of 17 cows has died; necropsy of a cow with hemoglobinuria, jaundice and focal necrotic hepatic foci has been performed
Late January–February 2024	Molecular and histopathological analyses confirmed *Clostridium haemolyticum* infection; six additional cow deaths occurred, totaling 23 fatal cases
End of February 2024	The outbreak ceased

## Discussion and conclusions

Bacillary hemoglobinuria is a disease of cattle with a geographic distribution dependent on soil characteristics and the presence of liver flukes metacercariae on grazing pastures. Poorly drained pastures and alkaline water pH favor the long-term survival of *C. haemolyticum* spores, which may persist for many years in the environment (Navarro et al. 2016).

The disease is considered endemic in many South American countries and in the western United States, although sporadic cases have been reported in various other countries worldwide. The historical geographic distribution of bacillary hemoglobinuria in southern South America includes southern Brazil, Uruguay, and Argentina (Schild 2007; Navarro et al. 2017; Micheloud et al. 2018). In the Brazilian state of Rio Grande do Sul, as well as in Uruguay the disease is mainly reported in floodplains, particularly in fields used for rice cultivation (Raymundo et al. 2014; Navarro et al. 2017; Micheloud et al. 2018; Santos et al. 2019). In the state of Santa Catarina, also in southern Brazil, outbreaks are most commonly seen after flooding events (Schild 2007).

To the best of our knowledge, the present report represents the first description of bacillary hemoglobinuria in the Central-West region of Brazil and the second report of the disease outside the southern states of Brazil (Melo et al. 2022). Similar to the previously reported case, in the tropical zone of Brazil (Melo et al. 2022), no gross and histologic findings characteristic of *F. hepatica* parasitism were found in the cow’s liver in our case. However, it is important to note that fascioliasis has previously been reported in cattle in the state of Goiás, based on individual reports and slaughterhouse data (Araújo et al. 2007; Aquino et al. 2018). Additionally, the parasitological method employed for detecting trematode eggs in feces is known to have low sensitivity (Micheloud et al. 2018).

Although under natural field conditions the disease is usually linked to the migration of *F. hepatica* larvae, several other causes of liver hypoxia have been listed, including necrotic foci induced by *Fusobacterium necrophorum*, parasitism by *Cysticercus tenuicollis*, trauma from hepatic biopsy, telangiectasia, and fatty hepatocellular degeneration (Janzen et al. 1981; Radostits et al. 2002; Navarro et al. 2017). In the present case, it was not possible to determine the initial lesion, nor evidences of tracts, scars or hematin deposition that could suggest any trematode migration, which could trigger an initial focus of anaerobiosis, leading to the germination of *C. haemolyticum* spores (Navarro et al. 2016). However the liver of the cow exhibited a concomitant multifocal hepatocellular degeneration, which could be either a pre-existing lesion derived from metabolic changes, hypoxia due to hemolytic anemia by other causes, or secondary to the manifestation of the disease. In this regard, pregnancy has been suggested as a potential cause of reduced liver blood flow, a hypothesis supported by the observed peak of bacillary hemoglobinuria cases during the winter months in Buenos Aires Province, Argentina, when cows are in the final stage of gestation. Ruminal expansion due to a fibrous diet is also presumed to cause hepatocellular hypoxia due to liver compression (Micheloud et al. 2018). *A. marginale*, which was detected in the spleen of the cow in our case, can also cause hepatocellular necrosis, secondary to centrilobular hypoxia in cattle (Fighera and Graça 2016). Those foci of hepatocellular necrosis could eventually elicit the germination of, *C. haemolyticum* spores. It is important to note, however, that after recovery from acute anaplasmosis, cattle can remain persistently infected by this rickettsial parasite (De Echaide et al. 1998; Reinbold et al. 2010). The role of the above-mentioned contributing factors remains under debate (Navarro et al. 2017; Micheloud et al. 2018).

Unlike the vast majority of reported cases, in which bacillary hemoglobinuria predominantly affects extensively raised beef cattle (Raymundo et al. 2014; Micheloud et al. 2018; Santos et al. 2019), the present report describes the disease in a dairy cow raised under semi-intensive conditions. The reason for the predominance of the disease in pasture-grazing cattle resides on the exposure of the animals to areas infested with liver flukes, including areas near bodies of water, especially during dry periods (Navarro et al. 2016).

There was no clear cause identified for the high mortality observed on the farm, as the majority of the affected cows had already died by the time of the visit. However, the available evidence suggests a multifactorial disease outbreak. It is plausible that the introduction of new cows to the herd without adopting quarantine measures could have played a critical role in the emergence of the outbreak (Radostits et al. 2002). The farmer also reported the indiscriminate intramuscular administration of drugs and vaccines using the same needle for both the newly introduced and resident cows (Radostits et al. 2002; Reinbold et al. 2010).

The main differential diagnosis of bacillary hemoglobinuria is infectious necrotic hepatitis caused by *Clostridium novyi* type B that also causes necrotic hepatic lesions containing bacillary colonies and thrombosis. A few gross findings can help to differentiate both conditions, including the usual absence of hemolytic changes and a tendency to form smaller and multiple foci of hepatic necrosis in infectious necrotic hepatitis (Navarro and Uzal 2016). In our case, besides all characteristic clinical and anamopathological findings of bacillary hemoglobinuria, molecular studies allowed the diagnosis to be stablished.

In the Central-West region of Brazil, the disease must also be differentiated from other causes of hemolytic anemia with hemoglobinuria and/or jaundice in cattle, including chronic copper poisoning (Martins et al. 2020), babesiosis (Rondelli et al. 2017) and toxic hemolytic anemia of unknown etiology (Rondelli et al. 2018). Leptospirosis could also cause jaundice and hemoglobinuria in cattle, however acute hemolytic disease by this agent affects mainly calves (Reis et al. 2017; Murillo et al. 2024). Postparturient hemoglobinuria should also be considered as a differential diagnosis in dairy cows presenting a hemolytic disease (Vine et al. 2006).

In this report, the diagnosis of bacillary hemoglobinuria in a dairy cow was supported by compatible gross and histopathological lesions and detection of *C. haemolyticum* DNA in liver tissue. *Fasciola hepatica* infection was not demonstrated by the methods used but could not be categorically excluded. We conclude that bacillary hemoglobinuria should be considered in the differential diagnosis of cattle with hemoglobinuria, jaundice, and focal necrotic hepatic lesions in the Central-West region of Brazil.

## Data Availability

Pathological and molecular data were generated and analyzed during the current study and are available from the corresponding authors upon reasonable request. The nucleotide sequences obtained were deposited in the GenBank database under the submission number SUB16180329.
